# Atypical Microbiological Feature of Infectious Endophthalmitis on Jeju Island: A 10-Year Study at a Single Tertiary Referral Center

**DOI:** 10.1155/2021/6620926

**Published:** 2021-03-02

**Authors:** Joong Hyun Park, Dong Yoon Kim, Ahnul Ha, Dae Joong Ma, Hye Jin Lee, Jinho Jeong, Jin Young Kim

**Affiliations:** ^1^Department of Ophthalmology, Jeju National University Hospital, Jeju National University School of Medicine, Jeju, Republic of Korea; ^2^Department of Ophthalmology, Chungbuk National University Hospital, Chungbuk National University College of Medicine, Cheongju, Republic of Korea; ^3^Department of Ophthalmology, Hallym University Kangnam Sacred Heart Hospital, Hallym University College of Medicine, Seoul, Republic of Korea

## Abstract

**Background:**

To analyze the microbiological causes of infectious endophthalmitis on an isolated island over ten years.

**Methods:**

A retrospective review of the medical records of 49 eyes clinically diagnosed with infectious endophthalmitis between January 2009 and December 2018 was done. The subjects were recruited from a single tertiary referral center on Jeju Island. The baseline characteristics of all subjects were investigated, and a culture examination was performed. The isolated pathogens were analyzed to determine how their microbiological features differed from those in regions with different geographical conditions.

**Results:**

Of the 49 eyes, causative microorganisms were identified in 27 eyes (55.1%). Bacteria were found in 24 cases and fungi in 3 cases. Among the exogenous causes, *Staphylococcus* species (*Staphylococcus aureus, S. epidermidis, and S. hominis*) were the most common pathogens (7 cases). *Achromobacter xylosoxidans* was the second most common causative pathogen (4 cases) followed by *Moraxella* species (3 cases). The most frequent endogenous origin was due to *Klebsiella pneumoniae* (6 cases). The subjects were divided into two groups according to the treatment results and analyzed for predisposing factors related to visual outcomes. The presence of diabetes mellitus (*P* = 0.038) and initial visual acuity (*P* ≤ 0.001) were significant predisposing factors for visual outcomes.

**Conclusion:**

The causative microorganisms of endogenous endophthalmitis on Jeju Island were not different from those reported previously. However, isolated exogenous microorganisms were different from those reported in other studies from inland areas. A high incidence and atypical clinical features of *Achromobacter xylosoxidans* and *Moraxella* in exogenous endophthalmitis were observed, reflective of the distinct climatic features of Jeju Island: high humidity and temperature. Therefore, considering the causative microorganisms of exogenous endophthalmitis, it may be assumed that the causative microorganisms of exogenous endophthalmitis and its clinical manifestations differ according to the region.

## 1. Introduction

Infectious endophthalmitis is one of the most fatal complications of ophthalmic diseases, characterized by severe intraocular infection originating from either exogenous or endogenous factors. In exogenous endophthalmitis, the causative organism directly invades the eye. While postcataract endophthalmitis and posttraumatic endophthalmitis constitute the majority of exogenous endophthalmitis seen worldwide, endophthalmitis can also occur in conjunction with intravitreal injections, keratitis, bleb after filtering surgery, and scleral buckle [1, 2]. Postoperative cases accounted for 40–80%, and posttraumatic cases showed an incidence of 2–15% out of the total endophthalmitis cases in countries like England, Australia, Korea, and Brazil, whereas the incidence of posttraumatic endophthalmitis was higher in developing countries including India, Egypt, and Thailand [1, 3–5]. Endogenous endophthalmitis is less common than exogenous endophthalmitis and is transmitted hematogenously from distant foci of infection within the body [6].

The clinical features of infectious endophthalmitis vary according to the causative microorganism [7, 8]. Therefore, identifying the causative organism through culture is critical. The frequency of causative microorganisms in endophthalmitis varies depending on the risk factors for endophthalmitis and its geographic locations. In postcataract endophthalmitis, *Staphylococcus* has been reported to be the most frequently isolated exogenous pathogen globally including in Korea [9–12]. In other studies, *Streptococcus viridans and Pseudomonas aeruginosa* were referred to as common exogenous pathogens of postcataract endophthalmitis [13–15]. In contrast, the fungus was found to be a common cause of postoperative endophthalmitis in tropical areas including India [16–18]. *Bacillus* species are known as major pathogens in posttraumatic endophthalmitis, causing fulminant infections with poor prognosis [19]. In a prospective study on postintravitreal anti-VEGF endophthalmitis in England, coagulase-negative *staphylococci* and *Staphylococcus epidermidis* presented in higher frequencies [20]. Keratitis can spread to cause endophthalmitis; a case series in Florida found that 53% of keratitis-related endophthalmitis was caused by molds contrary to an incidence of 17% of in a similar case series from New Jersey, assumed to be attributed to the high humidity of the Florida region [21, 22]. Endophthalmitis is sometimes known to occur after glaucoma filtering surgery, and blebs can be a source of infection. Blebitis and leakage from blebs are associated with progression to endophthalmitis, and *Streptococcus viridans* and *Staphylococcus pneumoniae* are the main causative pathogens [1]. There are few cases of endophthalmitis associated with scleral buckle [23, 24]. Meduri et al. [2] proposed that exposure to the scleral buckle could be a cause of endophthalmitis. The common pathogens of endogenous endophthalmitis in Western countries include *Staphylococcus aureus, Streptococcus pneumoniae, and Escherichia coli* [6]. In Asia, *Klebsiella pneumoniae* is the main source of endogenous endophthalmitis closely associated with liver abscesses [25]. As previous studies have indicated that the causative microorganisms may vary according to regional and climatic factors [7, 19, 26], it is rational to presume that exogenous pathogens originating from the environment are affected by a particular region's geoclimatic conditions. Jeju Island is located in the southernmost part of Korea surrounded by the ocean from all sides allowing speculation that the warm and humid climate may have allowed the spread of distinct causative microorganisms. As this study is the first report of infectious endophthalmitis on Jeju Island from a single tertiary referral center, this paper reflects on the clinical features and visual outcomes of exogenous and endogenous endophthalmitis in different climates in Korea over a period of ten years.

## 2. Materials and Methods

This study was approved by the Institutional Review Board of Jeju National University Hospital (IRB No. 2019-01-011) and was conducted in accordance with the Declaration of Helsinki. The primary objective was to report the microbiological features of infectious endophthalmitis on Jeju Island. The secondary objective was to determine the rationale for the distribution of causative organisms of infectious endophthalmitis, which is different from that of other regions in Korea.

### 2.1. Subjects

Patients with infectious endophthalmitis that occurred and was diagnosed on Jeju Island were included in this study. Based on the origin of infectious endophthalmitis, it was classified as either exogenous or endogenous. Endophthalmitis originating from a source within the body was defined as endogenous, and that due to direct inoculation of an organism from outside was defined as exogenous. Exclusion criteria were as follows: (a) patients referred from other regions; (b) patients with diseases mimicking infectious endophthalmitis (noninfectious uveitis, blebitis, sterile endophthalmitis after intravitreal injection or surgery); (c) patients with diseases (corneal opacity, progressed cataract [≥ moderate stage), glaucoma, and comorbid retinal diseases such as diabetic retinopathy, retinal vein or artery occlusion, and age-related macular degeneration) that could significantly affect visual acuity; and (d) patients with follow-up duration <6 months.

### 2.2. Baseline and Follow-Up Examination

Slit-lamp examination was performed to observe the clinical features, including eyelid swelling, conjunctival injection and discharge, anterior chamber cells and flare, fibrinous material, and hypopyon. Additionally, B-scan ultrasonography was performed when the fundus was invisible owing to severe vitreous haziness. Symptoms such as ocular pain, photophobia, and decreased visual acuity were also considered in the diagnosis of infectious endophthalmitis. Data including age, sex, systemic diseases, initial and final visual acuity, intraocular pressure, presence of hypopyon, culture results, number of intravitreal injections and vitrectomy, and treatment success rate were collected.

### 2.3. Culture Method

Aqueous humor was aspirated using a 30-gauge needle or a vitreous sample was obtained from the infusion line connected to a 25-gauge port after the patient underwent pars plana vitrectomy. The obtained samples were sent for culture and grown on blood agar, chocolate agar, MacConkey agar, and Sabouroud agar plates, and antibiotic sensitivity tests were performed.

### 2.4. Treatment Modality and Indication

Management included intravitreal injection (vancomycin 1.0 mg/0.1 mL, ceftazidime 2.25 mg/0.1 mL), topical fortified antibiotics, and intravenous or oral broad-spectrum antibiotics; intravitreal voriconazole (100 *μ*m/0.1 mL) was administered when fungal infection was suspected. Pars plana vitrectomy was performed for patients with (a) invisible fundus due to severe infection and inflammation, (b) no improvement after initial treatment, (c) visual acuity ≤ counting fingers, or (d) severe clinical features at the initial visit. After initiating treatment, the treatment regimen was modified according to the results of the antibiotic sensitivity test and culture.

### 2.5. Environmental Factors' Investigation

To compare the differences in environmental factors between Jeju Island and other inland areas, data from the Korea Meteorological Administration during the same study period (10 years, 2009–2018) in Korea were analyzed. The following environmental factors were investigated: average annual temperature, average annual precipitation, average annual relative humidity, and annual sunshine hours.

### 2.6. Data Analysis

The initial and final visual acuities were estimated through manifested refraction and converted to the logMAR scale for statistical analysis. The visual acuity of no light perception, light perception, hand motion, and counting fingers were set as 3.0, 2.5, 2.3, and 2.0, respectively [27]. Treatment failure was defined as final visual acuity ≥logMAR 1.0 or cases with evisceration due to uncontrolled inflammation and severe pain. Based on these criteria, we divided the subjects into two groups: treatment success group and treatment failure group. The predisposing factors that could influence visual outcomes were analyzed.

Statistical analyses were performed using SPSS version 23 (SPSS Inc., Chicago, IL, USA), and variables were compared using independent *t*-tests and Pearson's chi-square tests. Statistical significance was set at *P* value <0.05.

## 3. Results

### 3.1. Baseline Characteristics

A total of 49 eyes diagnosed as infectious endophthalmitis were included in the study at Jeju National University Hospital. The baseline characteristics of the subjects and the predisposing factors are summarized in Table 1. The culture-positive rate was 55.1% (27/49); 24 bacterial and 3 fungal causes were identified (Table 1). The causes of infectious endophthalmitis were 39 cases originated from exogenous factors, nine cases from endogenous factors, and one causative factor could not be identified. Cataract surgery was the most common cause of exogenous endophthalmitis (24/39, 61.5%), while other exogenous factors included trauma (8/39, 20.5%), other ophthalmic surgeries (4/39, 10.3%), and infectious keratitis (3/39, 7.7%) (Table 2). The most common cause of endogenous endophthalmitis was a liver abscess (7/9, 77.8%).

### 3.2. Isolated Microorganisms

Table 3 shows the microbial distribution, treatment, and treatment success rate of exogenous and endogenous endophthalmitis cases. Among the exogenous endophthalmitis cases, *Staphylococcus* spp. showed the highest frequency (7/20, 35.0%). As a solitary cause of exogenous endophthalmitis, *S. epidermidis* and *Achromobacter xylosoxidans* were the most common pathogens, with 4 cases each (4/20, 20.0%). They were followed by *Moraxella* species (3/20, 15.0%), *S. aureus* (2/20, 10.0%), *Pseudomonas aeruginosa* (2/20, 10.0%), and *Fusarium* species (2/20, 10.0%). The most common origin of endogenous endophthalmitis was *Klebsiella pneumoniae* (6/7, 85.7%), and one case of *Candida* species (1/7, 14.3%) was detected.

**Figure 1:**
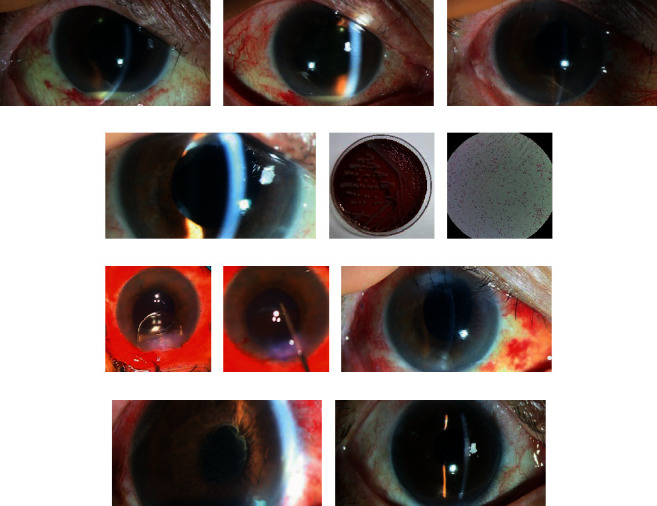
Representative cases of endophthalmitis caused by *Achromobacter xylosoxidans*. (a) The corneal edema, anterior chamber cell reaction, hypopyon, and pupillary membrane were found at the first visit. (b) In spite of pars plana vitrectomy and intravitreal injection, the endophthalmitis has aggravated. (c) Intraocular lens was removed and partial lens capsule was removed by the outcome and quiet state persisted. (d) After ciliary sulcus fixation of the intraocular lens, cellular reactions have recurred. (e) The Gram-negative rod *Achromobacter xylosoxidans* was revealed from blood agar plate culture. (f) Microphotograph of *Achromobacter xylosoxidans* with gram staining (×1000). (g) Surgical procedure showing removal of the intraocular lens. (h) The remnant lens capsule is being removed completely using microforceps. (i) After additional vitrectomy, repeated intraocular lens removal, and en bloc delivery of the lens capsule, recurrence has stopped thereafter. (j) Another patient showed the anterior chamber reaction and severe pupillary fibrotic membrane from the initial presentation. (k) Completely healed state after repeated intravitreal injections and vitrectomies, including en bloc delivery of the lens capsule.

**Figure 2:**
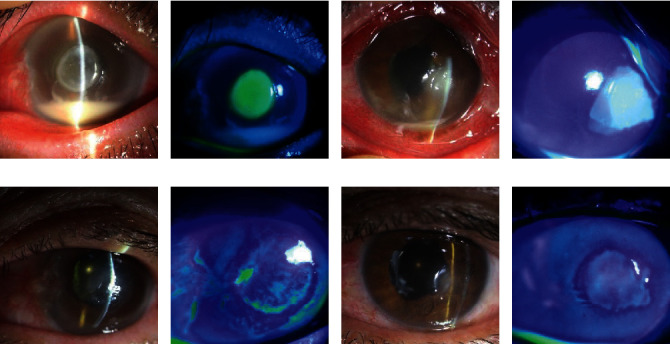
Representative cases of endophthalmitis caused by *Moraxella* species. In both cases, infectious keratitis was the predisposing factor. (a, b) Corneal edema, central infiltration, and epidefect were observed along with significant hypopyon at the initial visit. (c, d) Regressed central infiltration and epidefect after intravitreal antibiotic injection. (e, f) Conjunctival chemosis and injection, 4°/c corneal infiltration with epidefect, pupillary membrane, and hypopyon were seen. (g, h) Pars plana vitrectomy and intravitreal antibiotic injection were performed, and the endophthalmitis has cleared up.

About 71.4% (5/7) cases with *Staphylococcus* species were successfully treated while 100% of cases with *Achromobacter xylosoxidans* were successfully treated despite the high frequency of pars plana vitrectomy (12 times) and intravitreal injection (49 times). However, *Klebsiella pneumoniae, Moraxella* species, and fungal species showed relatively poor response to treatment (33.3%) (Table 3). Figures 1 and 2 show representative cases of *Achromobacter xylosoxidans* and *Moraxella* species endophthalmitis.

### 3.3. Climatic Feature

**Figure 3:**
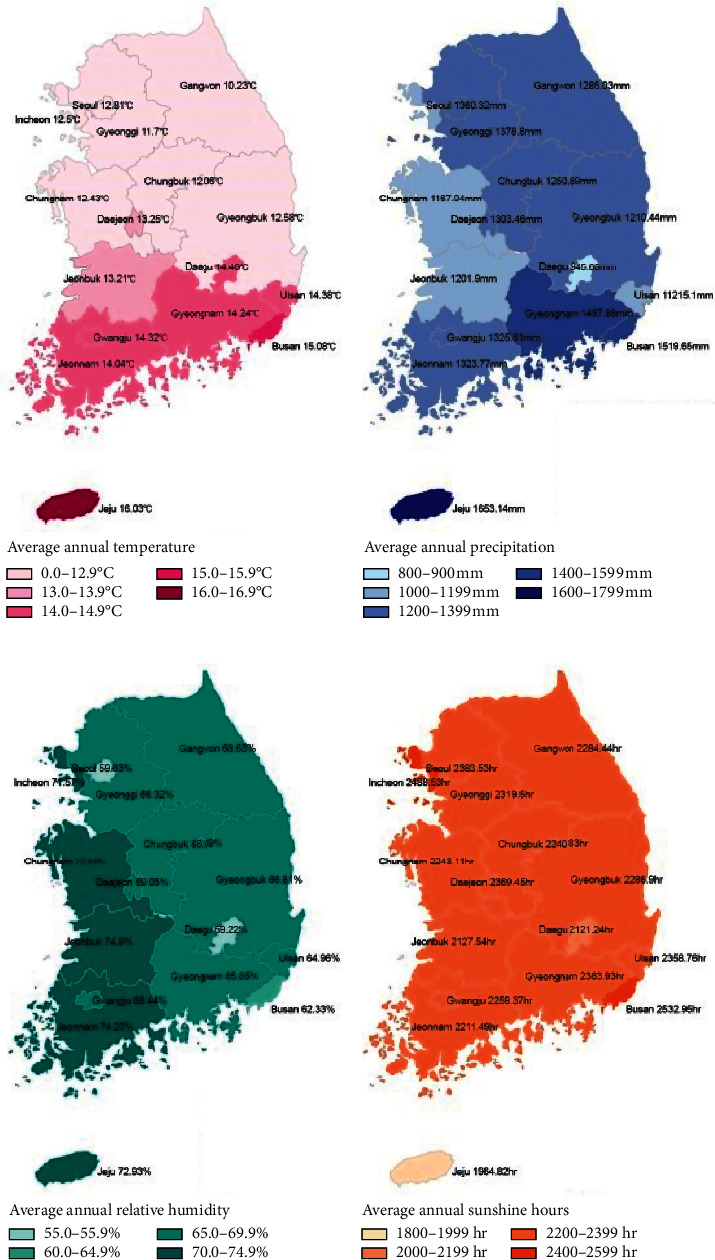
The average temperature, the annual precipitation, the average humidity, and the annual sunshine hours for 10 years (2009–2018) in South Korea. The graph showed that Jeju Island had the highest average temperature (1^st^ place), the highest annual precipitation (1^st^ place), higher average relative humidity (4^th^ place), and the lowest annual sunshine hours (1^st^ place) for 10 years in the country.

The environmental factors of Jeju Island using data from the Korea Meteorological Administration for 10 years (2009–2018) were evaluated. Jeju Island showed the highest average annual temperature and average annual precipitation (top place), higher average annual relative humidity (fourth place), and the lowest annual sunshine hours (first place) over 10 years in Korea (Figure 3).

### 3.4. Clinical Outcomes

The treatment success and failure rates were compared and summarized in Table 4. The mean age in the treatment success and treatment failure groups were 62.90 ± 17.90 years and 72.13 ± 13.67 years, respectively. In both groups, there were more males than females, and the male-to-female ratio in the treatment success and treatment failure groups was 17 : 15 (53.1% males) and 9 : 8 (52.9% males), respectively. The culture positivity rate in the treatment success group was higher (40.6%) than that in the treatment failure group (35.3%); however, the difference was not significant (*P* = 0.715). Hypopyon was observed more commonly in the treatment failure group (66.7%) than in the treatment success group (56.3%); however, no significant difference was found between the two groups (*P* = 0.566). The two groups were compared according to the number of treatments, such as intravitreal injection and/or vitrectomy. The mean number of intravitreal injections was 3.77 ± 4.95 in the treatment success group, and 2.80 ± 1.93 in the treatment failure group, and this difference was statistically insignificant (*P* = 0.472). There were no significant differences in the number of vitrectomies (*P* = 0.153).

There were two main predisposing factors related to the visual outcomes of infectious endophthalmitis in the comparison between the two groups. First, the initial best-corrected visual acuity (BCVA) was the most important factor in determining the final visual outcome. The initial visual acuity presented as logMAR in the treatment success and treatment failure groups was 1.71 ± 0.76 and 2.47 ± 0.23 (*P* ≤ 0.001), respectively. Moreover, diabetes mellitus was relatively common in the treatment failure group (47.1%), which had a higher risk than the treatment failure group did (*P* = 0.038).

## 4. Discussion

The primary finding of this study is a unique distribution of exogenous pathogens in endophthalmitis, which is different from most previous reports. A previous study reported significantly higher incidences of *Pseudomonas* and *Aspergillus* species as causes of postoperative endophthalmitis in India with tropical climatic conditions [17]. Furthermore, Ramos et al. [28] revealed an association between *Pseudomonas aeruginosa* and high humidity, temperature, and precipitation. In addition, some studies found that the frequency of Gram-negative bacteria such as *Enterobacter cloacae and Klebsiella pneumoniae* increased with an increase in temperature [29, 30]. Jeju Island has a warm and humid climate throughout the four seasons, unlike other inland regions in Korea. Therefore, this study focused on determining how this unique feature of Jeju Island might contribute to the differences in microbial features from other regions in Korea.

Endogenous endophthalmitis is known to be largely affected by individual chronic underlying diseases and medical conditions such as diabetes mellitus, infectious endocarditis, and liver cirrhosis [6]. In Western countries including the United States and Europe, Gram-positive bacteria and fungi are common, whereas *Klebsiella pneumoniae* is the most common cause of endogenous endophthalmitis in East Asia, including Korea [31]. This variation, which has not yet been revealed, is thought to be associated with intravenous drug abuse in Western countries, genetic differences between racial groups, and a high incidence of cholangiohepatitis in East Asia [31, 32]. This study showed similar results to previous reports in Korea showing a predominance of *Klebsiella pneumoniae* as the causative microorganism in endogenous endophthalmitis [25, 31, 32].

The Endophthalmitis Vitrectomy Study indicated that *Staphylococcus epidermidis* was the most commonly isolated exogenous pathogen followed by *Staphylococcus aureus*, *Streptococcus* species, and *Enterococcus* species [33]. Other studies have also shown *Staphylococcus epidermidis* to be the most common pathogen [9, 34] while Torabi et al. [13] revealed that *Streptococcus viridans* was the most common pathogen. Kim et al. [14] and Dave et al. [15] reported that *Pseudomonas aeruginosa* was the most commonly isolated pathogen. Unlike previous studies, this study's results demonstrated a high frequency of *Achromobacter xylosoxidans* (4/20, 20.0%) and *Moraxell*a species (3/20, 15.0%) as causes of exogenous endophthalmitis.

*Achromobacter xylosoxidans* is a very rare cause of infectious endophthalmitis. This microorganism adapts well in humid and warm climates [35]. According to Holmes et al., several strains of *Achromobacter xylosoxidans* were introduced, and the sources of detection included blood, urine, sputum, swimming pools, and water tanks. Of the 14 strains observed, 13 presented positive growth at 37°C except for one that was incubated at room temperature [36]. Moreover, a high incidence of *Moraxella* species was also found, which is a unique aspect of this study. *Moraxella* species are normal flora microorganisms known to flourish under moist and high-temperature conditions, and Larsen et al. [37] reported that the colonies tended to spread at 37°C in a moist chamber. This pathogen, with low virulence, exists in the upper respiratory tract, genitourinary tract, and conjunctiva, known to cause ocular diseases such as infectious keratitis, conjunctivitis, and endophthalmitis [38, 39]. According to a Korean study on infectious keratitis, infection by *Moraxella* species was only observed in 0.3% of the total 689 cases [40], and only a few cases of infectious endophthalmitis associated with a bleb at the delayed onset have been described [41, 42]. These characteristics of *Achromobacter xylosoxidans* and *Moraxella* species match the climate of Jeju Island. It is an isolated island surrounded by the ocean, with high all-year-round temperature and humidity. In addition, the sunlight exposure time was the lowest in Korea, which is the optimum condition for the growth of these bacteria (Figure 3).

*Achromobacter xylosoxidans* displayed a notable treatment process compared to the other pathogens detected in our study. Few cases of severe recurrent endophthalmitis caused by *Achromobacter xylosoxidans* have been reported [43]. Owing to the characteristics of *Achromobacter xylosoxidans* in creating a biofilm, and remaining in the capsular bag and intraocular lens, a high tendency of recurrence was observed despite simple pars plana vitrectomy and intravitreal antibiotic injections [44, 45]. Two cases in the current study underwent pars plana vitrectomy six and four times each and were administered with intravitreal injections 23 and 18 times, respectively. After several recurrences, radical capsulectomy was performed along with intraocular lens removal to terminate the episode. For the other two cases of infectious endophthalmitis caused by *Achromobacter xylosoxidans*, pars plana vitrectomy, radical capsulectomy, and intraocular lens removal were the initial treatment modalities, and complete remission was achieved (Table 3). The average final BCVA was 0.43, which ranged from 0.2 to 0.7, and all four cases were successfully treated.

There has been evidence that humidity and temperature were associated with the incidence of *Pseudomonas aeruginosa* [28], and an increasing rate of Gram-negative species was found at high temperatures [29, 30]. Unlike endogenous endophthalmitis, which arises from a specific focus of infection in the body, the source of exogenous endophthalmitis mainly originates from periocular normal flora. Being exposed to the outside environment, the incidence and distribution of normal flora could possibly be affected and changed by the humid and warm climate of Jeju Island, thus justifying the rare presentation of *Achromobacter xylosoxidans and Moraxella* species in this study.

Considering the predisposing factors of infectious endophthalmitis in our study, cataract surgery was the most common cause (24/49, 48.9%) followed by endogenous cause (18.4%) and trauma (16.3%). Of the eight cases of trauma-induced infectious endophthalmitis, six patients were injured during farm work; therefore, it can be presumed that the higher proportion of traumatic causes was due to the relatively large number of agricultural workers on Jeju Island. In contrast, the most common cause of endogenous endophthalmitis was a liver abscess. This result is similar to that of many previous studies in East Asia and Korea [25, 31, 32].

Factors associated with final visual acuity have been reported to be initial visual acuity, diabetes mellitus, the presence of microorganisms, and hypopyon in previous studies [33, 46, 47]. Ho et al. [9] identified culture-negativity as a positive prognostic factor for better visual acuity, under the assumption that more bacterial loads may be present in culture-positive cases. Diabetes mellitus and initial visual acuity were the only factors associated with the treatment success rate in this study.

To the best extent of the author's knowledge, this is the first study on endophthalmitis on Jeju Island. In addition, the data collection over ten years from a single tertiary center could aid in strengthening the analysis of the features of endophthalmitis on Jeju Island. Moreover, it can be proposed that exogenous endophthalmitis might be affected by environmental factors around the causative pathogens. This study has some limitations. First, Jeju Island is a popular tourist spot, and some of the subjects were from other regions that deteriorated the distinctiveness of the microbiome in endophthalmitis on Jeju Island. Furthermore, owing to its retrospective design, some of the patient's data were missing or distorted. This report showed the environmental effects of exogenous pathogens; however, the control groups from another region with different geographic locations were not included. Further data collection and comparative investigation of districts with different climatic conditions might yield more significant results.

## 5. Conclusions

This study investigated and reported the microbiology, incidence, and clinical features of Jeju Island for the first time. This decade-long study from a single tertiary center demonstrated atypical distribution in microorganisms of exogenous endophthalmitis, distinguishable from most previous studies. High incidences of rare pathogens as causes of endophthalmitis were detected, and the climatic features of Jeju Island have met the thriving conditions of these pathogens. Endophthalmitis is an ophthalmic emergency, and that can deteriorate ocular structures in a short time in the absence of accurate diagnosis and appropriate treatment. If unfavorable progress is observed despite adequate treatment, atypical pathogens of endophthalmitis regarding climatic and geographic features of the region should be suspected. These findings will be of particular interest to ophthalmologists and will contribute to the future treatment of infectious endophthalmitis on Jeju Island.

## Figures and Tables

**Table 1:** Baseline characteristics of patients in this study.

Characteristics	
Age (years)	65.98 ± 17.03^a^
Sex (male: female)	26 : 23

Underlying disease, *n* (%)	
Diabetes	12 (24.5)
Hypertension	36 (73.5)

Initial BCVA, LogMAR	0.39 ± 0.21^a^

Culture-positive, *n*/total (%)	27/49 (55.1)
Bacteria, *n* (%)	24 (88.9)
Fungus, *n* (%)	3 (11.1)

BCVA = best collected visual acuity. LogMAR = logarithm of the minimum angle of resolution. ^a^ Values are presented as mean ± standard deviation.

**Table 2:** Causative factors in infectious endophthalmitis.

Factors	No. of identified cases/total cases (%)
Exogenous	19/39 (48.7)
Cataract surgery	13/24 (54.2)
Trauma	2/8 (25.0)
Other surgery	2/4 (50.0)
Infectious keratitis	2/3 (66.7)

Endogenous	7/9 (77.8)
Liver abscess	6/7 (85.7)
Septic pneumonia	1/1 (100)
Urinary tract infection	0/1 (0)

Unknown	1/1 (100)
Total	27/49 (55.1)

**Table 3:** Identified organisms and treatment results in exogenous and endogenous endophthalmitis.

Causative organism			No.	The number of		
				PPV/EVI	Injections	Success (%)
Exogenous			20			
G (+) bacteria	*Staphylococcus* species	*Staphylococcus epidermidis*	4	3/1	8	3 (75.0)
		*Staphylococcus aureus*	2	2/0	3	1 (50.0)
		*Staphylococcus hominis*	1	2/0	4	1 (100)
	*Achromobacter xylosoxidans*		4	12/0	49	4 (100)
	*Streptococcus* species	*Streptococcus oralis*	1	2/0	6	0 (0)
		*Streptococcus intermedius*	1	1/0	2	1 (100)
G (−) bacteria	*Moraxella* species		3	3/1	6	1 (33.3)
	*Pseudomonas aeruginosa*		2	1/2	3	0 (0)
Filamentous	*Fusarium* specie*s*		2	2/1	8	1 (50.0)

Endogenous			7			
G (−) bacteria	*Klebsiella pneumoniae*		6	6/0	10	2 (33.3)
Yeast	*Candida* specie*s*		1	1/0	3	0 (0)
	Total		27			14 (51.9)

PPV = pars plana vitrectomy. EVI = evisceration.

**Table 4:** The comparison between treatment success and treatment failure groups.

Variables	No. of cases (%)		
	Treatment success (32 eyes)	Treatment failure (17 eyes)	*P*-value
Age	62.90 ± 17.90	72.13 ± 13.67	0.086^a^
Sex			0.990^b^
Male	17 (53.1)	9 (52.9)	
Female	15 (46.9)	8 (47.1)	

Diabetes mellitus			0.038^c^
Yes	5 (15.6)	8 (47.1)	
No	27 (84.4)	9 (52.9)	

Hypertension			0.801^b^
Yes	12 (37.5)	7 (41.2)	
No	20 (62.5)	10 (58.8)	

Hypopyon			0.566^b^
Yes	18 (56.3)	11 (66.7)	
No	14 (43.7)	6 (35.3)	

Culture results			0.715^b^
positive	13 (40.6)	6 (35.3)	
negative	19 (59.4)	11 (64.7)	

Initial BCVA (log MAR)	1.71 ± 0.76	2.47 ± 0.23	≤0.001^a^

Treatment number			
Vitrectomy	1.42 ± 1.30	0.85 ± 0.80	0.153^a^
Intravitreal injection	3.77 ± 4.95	2.80 ± 1.93	0.472^a^

PPV = pars plana vitrectomy. BCVA = best-corrected visual acuity. ^a^ Independent *t*-test; ^b^ Pearson's chi-square test; ^c^ Fisher's exact test.

## Data Availability

The datasets included in this study are available from the corresponding author upon reasonable request.
